# Interstitial ectopic pregnancy diagnosis by three-dimensional ultrasound and its laparoscopic management: A case report

**DOI:** 10.18502/ijrm.v17i12.5801

**Published:** 2019-12-30

**Authors:** Firoozeh Ahmadi, Fattaneh Pahlavan, Fariba Ramezanali, Farnaz Akhbari

**Affiliations:** ^1^ Department of Reproductive Imaging, Reproductive Biomedicine Research Center, Rayan Institute for Reproductive Biomedicine, ACECR, Tehran, Iran.; ^2^ Department of Endocrinology and Female Infertility, Reproductive Biomedicine Research Center, Rayan Institute for Reproductive Biomedicine, ACECR, Tehran, Iran.

**Keywords:** Pregnancy, Ectopic, Diagnostic, Ultrasound, Laparoscopic assisted surgery.

## Abstract

**Background:**

Interstitial Ectopic Pregnancy (IEP) is an uncommon type of ectopic pregnancy with the risk of rupturing and bleeding. The incidence of IEP is about 2-4% of all EPs. The diagnosis and management are challenging. We present a well-timed and managed case of IEP.

**Case:**

The case was a 37-yr-old woman presented at the Royan Institute with a chief complain of sudden onset of pelvic pain and moderate vaginal bleeding, three weeks after her positive pregnancy test. She had got pregnant with in-vitro fertilization procedure. She was admitted for a two-dimensional ultrasound (2DUS). The 2DUS findings showed a gestational sac with live embryo and yolk sac which was located high in the fundus and eccentric to the endometrium. The suspicion of IEP rose after the 2DUS findings, the confirmation of further diagnosis was then done by three-dimensional ultrasound, and the treatment was done by laparoscopy. The patient underwent laparoscopic left corneal resection. She was discharged after two days and her β-hCG achieved complete resolution ( < 5 mIU/mL) after two weeks' follow-up.

**Conclusion:**

According to the life-threatening complications that are associated with IEP, acquaintance and suspicion about IEP is important. Specified information that obtained by three-dimensional ultrasound could be useful for exact locating and detection.

## 1. Introduction

Interstitial ectopic pregnancy (IEP) is a type of ectopic pregnancy that occurs in the interstitial or intramural segment of fallopian tubes. The interstitial part is the proximal portion of the fallopian tube (1). The incidence of IEP is about 2-4% of all ectopic pregnancies (EPs) (2). Common etiologies are: a previous EP, in-vitro fertilization, pelvic inflammatory disease, and previous ipsilateral salpingectomy (3). The diagnosis and management are challenging (4). In interstitial pregnancy, the risk of hemorrhage and bleeding is higher than the other types of EP and can occur at any time in early pregnancy, so early diagnosis of IEP is crucial here (5). Ultrasonography especially three-dimensional ultrasound (3DUS) facilitates an early diagnosis and increases the success of conservative management for the prevention of rupture in most cases of IEP (6). Here we present a case of IEP with an aim to note the detection steps in favor of its facile management.

## 2. Case Report

**Figure 1:**
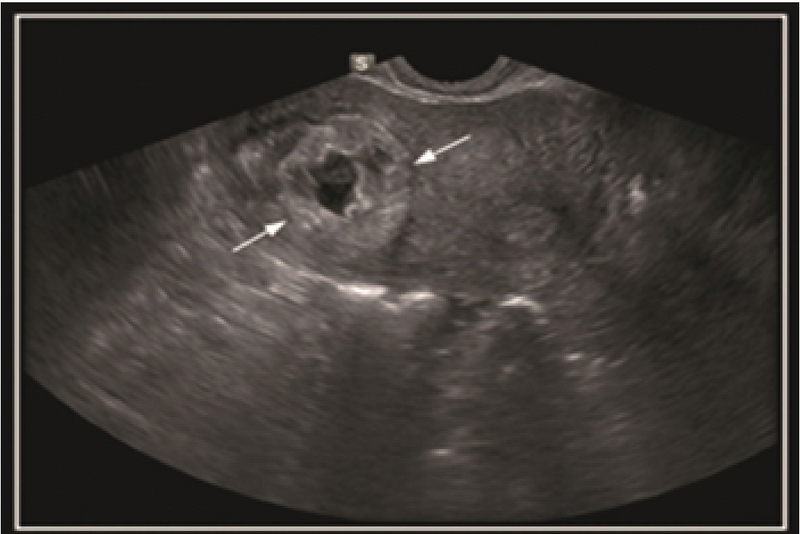
Interstitial pregnancy. Transverse transvaginal ultrasound image shows an eccentric gestational sac with an echogenic wall (arrows) partially surrounded by myometrium.

A 37-yr-old woman presented at the Royan Institute with a chief complain of sudden onset of pelvic pain. She had a history of 10 yr of infertility and hypothyroid. Her past history of surgery was diagnostic laparoscopy, hysteroscopy, and myomectomy. She became pregnant with in-vitro fertilization procedure. Three weeks after a positive pregnancy test, she came to the Royan Institute with sudden pelvic pain. Although she had moderate vaginal bleeding, she was hemodynamic ally stable and her laboratory test was normal. The β-Human Chorionic Gonadotropin (β-HCG) level on admission was 6550 mIU/mL, so she was admitted for two-dimensional ultrasound (2DUS). The 2DUS findings showed a gestational sac (GS) with live embryo and yolk sac which was located high in the fundus and eccentric to the endometrium (Figure 1). The suspicion of IEP was raised by the 2D ultrasonography findings.

**Figure 2:**
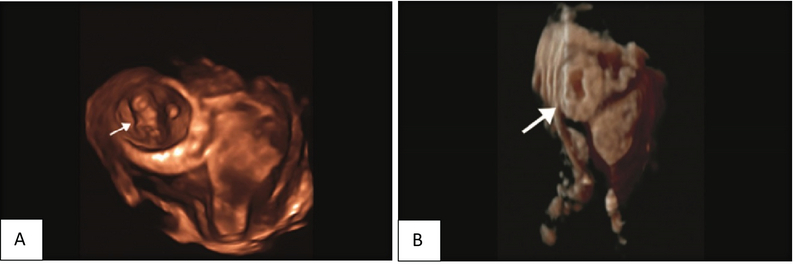
(A) Interstitial pregnancy. 3D-reconstructed image (coronal view) - gestational sac with the live embryo (arrow) located outside the endometrium and surrounded by myometrium in the right interstitial area. (B) Interstitial pregnancy (arrow). 3 Deconstructed image (coronal view).

Next, images by the 3DUS were obtained with the Medison A30 machine and the findings were reported as follows: GS with regular margin was seen at the left fundal portion of the uterus which was surrounded by myometrium. Also, the decidual reaction of endometrium was seen in the uterine cavity. Fetal pole and yolk sac could be detected in the GS. Gestational age was about six weeks and five days (Figure 2 A, B).

Based on the patient's condition, it seemed that the case would be managed laparoscopic ally. The patient underwent laparoscopic left corneal resection. There were adhesions around the uterus due to the previous myomectomy and all of the adhesions were resected.

Harmonic scalpel used for fallopian tube. Thickness and vascularity were seen in the junction of the uterine and fallopian tube. Bleeding was coagulated with bipolar cautery. Hemostasis was secured with bipolar cautery and the tube removed.

**Figure 3:**
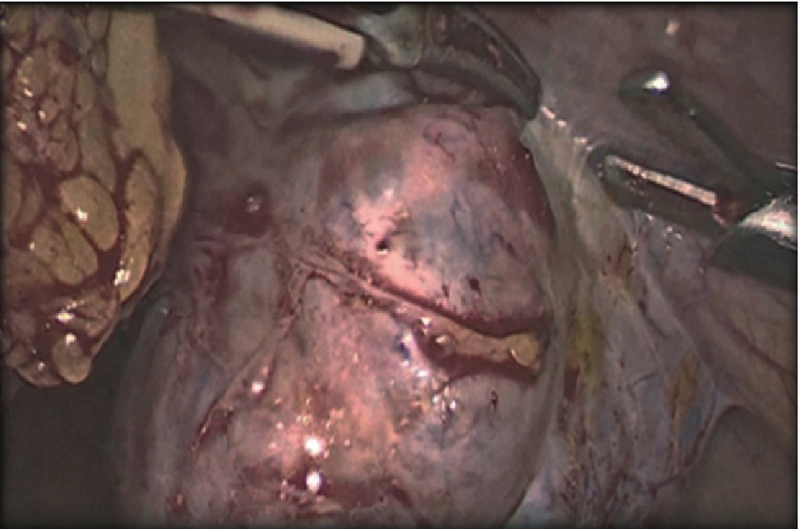
Laparoscopic view of the interstitial pregnancy.

The removed tube was put in anterior cul-de-sac and the corner of uterus was stich with 1-0 vicrryl. (Figure 3).Then the specimen was removed with adobos and was saved for pathology examination. Finally, the gas existed through umbilical trocar. All trocars were removed under the vision, the incision were closed with a Monocryl. The procedure lasted 2 hr. Considering that pre- and postoperative hemoglobin and another hemodynamic index of the patient were normal, there was no need for blood transfusion. The patient's postoperative course was uneventful. She was discharged after two days and her β-hCG achieved complete resolution ( < 5 mIU/mL) after two weeks' follow-up.

### Ethical Consideration

A written consent form was obtained from the patient for presenting this case.

## 3. Discussion

An IEP is a rare form of EP which is difficult to diagnose and may be confused with intrauterine pregnancy. Despite technical advances, the diagnosis of IEP remains difficult. IEP may be indistinguishable from other types of EP by 2DUS, therefore further evaluation by 3DUS is recommended in this situation. 3DUS makes it possible to detect the intramural portion of the fallopian tubes more clearly (Figure 3) (7).

Coronal plane in the 3DUS improved the spatial orientation of EP in relation to uterine cavity obtained in sonography. Implantation occurs in the interstitial portion of the fallopian tube which has more elasticity potential than another part of the fallopian tube, so this portion could be extended without any symptom typically (8, 9), hence it could be misdiagnosed in the early scan. IEP occurs at a vascularized portion of the uterine so the probability of life-threatening bleeding should be considered. If the rupture occurs, severe bleeding will happen and it is life-threatening for the patient (5). So early diagnosis by 2DUS and 3DUS as a non-invasive initial approach to diagnosis is very crucial here (9).

**Figure 4:**
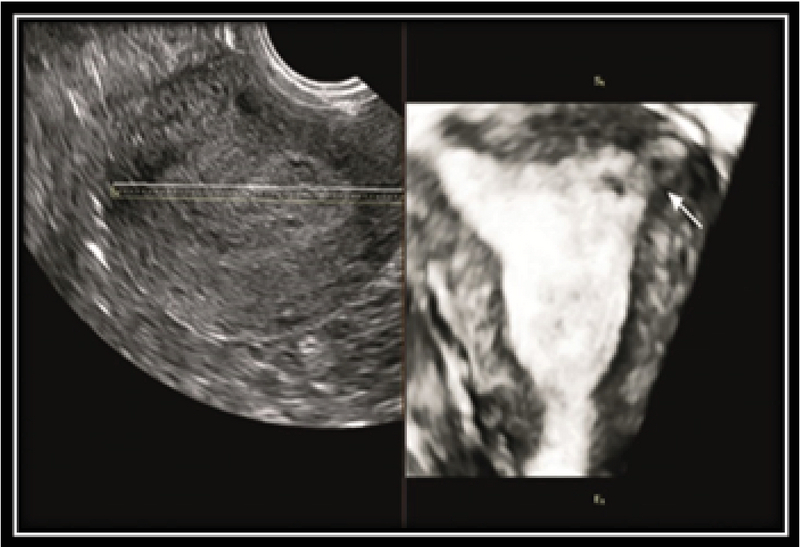
3DUS detect intramural portion of early pregnancy in the fallopian tubes clearly (arrow).

**Figure 5:**
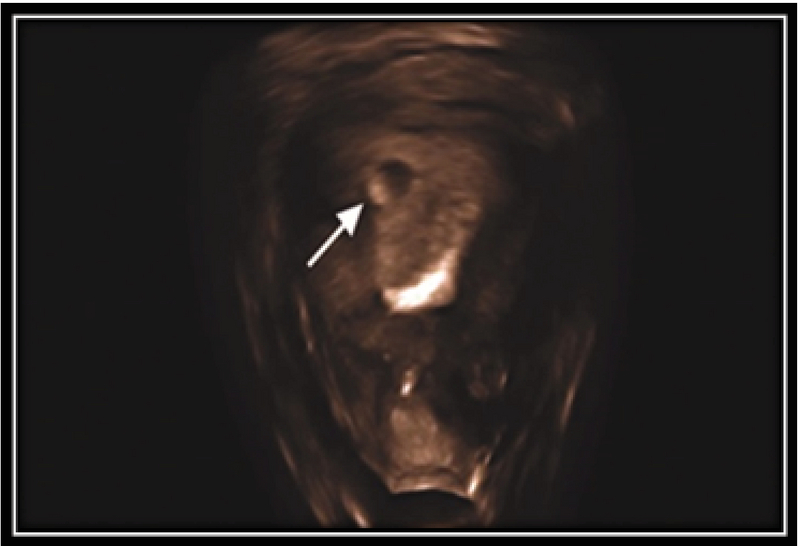
Intrauterine pregnancy: three-dimensional sonogram of the uterus shows the eccentric sac located within the endometrium (arrow).

The most important finding in IEP are: 1- an empty uterine cavity without gestational sac, 2- a chorionic sac which is take placed in lateral corner of uterine (the distance more than 1 cm from uterine cavity), 3-endometrial thickness less than 5 mm.

and a thin myometrium surrounding the GS ( < 5mm) (10). Many studies demonstrate the usefulness of 3DUS in the diagnosis of interstitial pregnancies (11). 3DUS showed clearly the intramural portion of the fallopian tube and has more sensitivity than 2DUS in the diagnosis of IEP. In a normal pregnancy, GS is usually in the lateral portion of the uterus early in gestation (Figure 4, 5).

It is remarkable that in advance gestational age of such case, the gestational sac may locate in fundus which misguides the detection. This is referred to interstitial line sign (an echogenic line from the endometrium to an EP) which is the best diagnostic clue (6, 12).

On the other hand, the IEP must be diagnosed with “septate or bicornuate uterus and from fibroids or myometrial contractions” (13). Laparoscopy has the diagnostic and treatment value in our case. Sharma *et al*. reported that laparoscopy has both diagnostic and treatment advantage in their case report (7).

## 4. Conclusion

Recently, using ultrasound and clinical expertise, delay diagnosis of IEP became less frequent. The progress in 3D TVUS and its coronal view makes it a precise diagnostic tool for the early diagnosis of IEP and differentiation of it from another type of pregnancy (11, 14).

##  Conflict of Interest

All contributing authors declare no conflict of interest.
